# Independent associations of childhood and current socioeconomic status with risk of self-reported doctor-diagnosed arthritis in a family-medicine cohort of North-Carolinians

**DOI:** 10.1186/1471-2474-14-327

**Published:** 2013-11-20

**Authors:** Antoine R Baldassari, Rebecca J Cleveland, Leigh F Callahan

**Affiliations:** 1 Thurston Arthritis Research Center, School of Medicine, University of North Carolina at Chapel Hill, Chapel Hill, NC, USA; 2 Department of Medicine and Social Medicine, University of North Carolina at Chapel Hill, Chapel Hill, NC, USA

**Keywords:** Arthritis, Lifecourse, SES, Health disparities

## Abstract

**Background:**

Associations of socioeconomic status (SES) with the prevalence of various forms of arthritis are well documented. Increasing evidence suggests that SES during childhood is a lasting determinant of health, but its association with the onset of arthritis remains unclear.

**Methods:**

Cross-sectional data on 1276 participants originated from 22 family practices in North-Carolina, USA. We created 4-level (high, medium, low, lowest) current SES and childhood SES summary scores based on parental and participant education, occupation and homeownership. We investigated associations of individual SES characteristics, summary scores and SES trajectories (e.g. high/low) with self-reported arthritis in logistic regression models progressively adjusted for race and gender, age, then BMI, and clustered by family practice.

**Results:**

We found evidence for independent associations of both childhood and current SES with the reporting of arthritis across our models. In covariate-adjusted models simultaneously including current and childhood SES, compared with high SES participants in the lowest childhood SES category (OR = 1.39 [95% CI = 1.04, 1.85]) and those in the low (OR = 1.66 [95% CI = 1.14, 2.42]) and lowest (OR = 2.08 [95% CI = 1.16, 3.74]) categories of current SES had significantly greater odds of having self-reported arthritis.

**Conclusions:**

Current SES and childhood SES are both associated with the odds of reporting arthritis within this primary-care population, although the possibly superseding influence of existing circumstances must be noted. BMI was a likely mechanism in the association of childhood SES with arthritis onset, and research is needed to elucidate further pathways linking the socioeconomic environment across life-stages and the development of rheumatic diseases.

## Background

Arthritis encompasses a diverse family of chronic disorders of uncertain etiology, characterized by inflammatory pain and joint degradation. Contrary to the common perception of arthritis as an unavoidable symptom of aging, preventable risk-factors including excess body mass and musculoskeletal injuries play a major role in the onset of the disorder [1]. The importance of modifiable risk-factors is underscored by marked socioeconomic disparities in disease prevalence for self-reported arthritis [2,3], which includes all disease subtypes, and for specific forms, including osteoarthritis (OA) [4,5] and rheumatoid arthritis (RA) [6,7]. How socioeconomic status (SES) influences the onset of arthritis remains unclear, with socioeconomically patterned health-behaviors suspected as possible pathways [8,9].

Epidemiological studies have increasingly emphasized the lasting health impact of early-life circumstances [10-12]. While research on the relevance of childhood SES in arthritis remains at an early stage, there is mounting evidence for plausible mechanisms. Childhood SES has for instance been associated with behavioral and environmental risk-factors [13-15] and with biological changes increasing vulnerability to inflammatory disorders [16]. Furthermore, the experience of childhood adversity, such as physical abuse or parental addiction, has been convincingly associated with arthritis onset in later life [17,18].

The limited literature on early SES and self-reported arthritis so far includes the findings that low parental income and low-status paternal occupation are, respectively, associated with a higher risk of reporting an arthritis disorder among middle-aged and elderly individuals. [19,20]. While the relationship between childhood SES and the onset of RA has been comparatively more researched, its independence from later socioeconomic characteristics remains uncertain [21-24]; notably, Parks and colleagues reported that a low SES during childhood was associated with a greater risk for RA among women from a US national cohort, provided that SES remained low across the life-course [24].

In previous cross-sectional analyses on a primary-care cohort of North-Carolinians, our group found current SES to significantly influence the odds of having self-reported doctor-diagnosed arthritis [3], and we recently reported independent associations of childhood and current SES with physical health outcomes among those participants [25]. This current study aims to investigate independent and combined associations of current and childhood SES with the odds of having self-reported arthritis within that same primary-care population.

## Methods

### Sample

Data originated from the Individual and Community Social Determinants of Arthritis Outcomes study (SODE), where phone surveys were administered to 4442 eligible participants in the North Carolina Family Medicine Research Network (NC-FM-RN), in 2004 and 2006. The NC-FM-RN is a network of 22 primary-care providers in North Carolina selected to represent the geographic and racial/ethnic diversity of North Carolina, and is described in greater detail in the literature [26].

**Figure 1:**
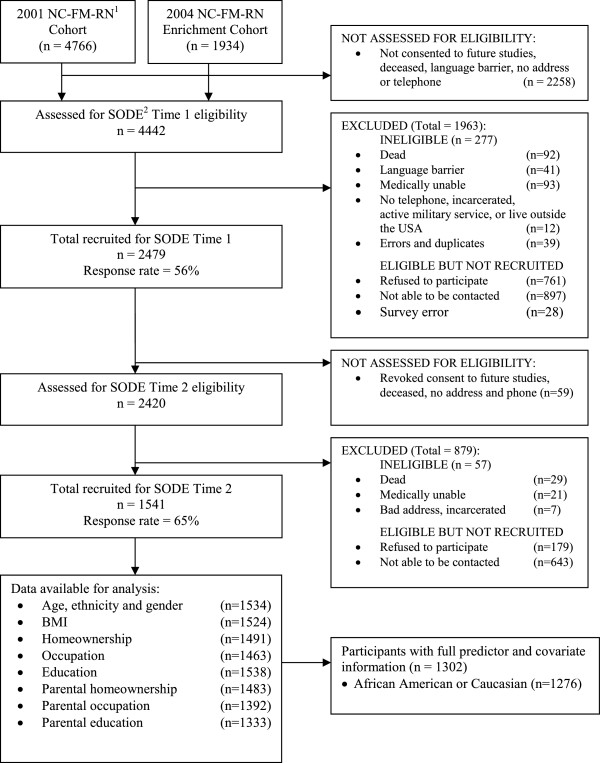
Flowchart of participants from the NC-FM-RN into the current study.

The first phone survey inquired about demographics, health status, attitudes and beliefs, chronic health conditions, and perceptions of neighborhood environment (n = 2479, response rate 56%), and the second extended to socioeconomic characteristics during childhood (n = 1541, response rate 62%). This study focuses on Caucasian and African-American SODE respondents who provided all relevant sociodemographic and health information (n = 1276, 83%). Individuals reporting their ethnicity as American Indian (n = 10), Asian (n = 4), Hawaiian or Pacific Islander (n = 1), or other (n = 11) were excluded from our analyses due to their small number. The flow of participants from the NC-FM-RN to the current study is detailed in Figure 1. All study materials and methods were approved by the University of North Carolina at Chapel Hill Biomedical Institutional Review Board.

### Measures

#### Self-reported arthritis

Self-reported doctor-diagnosed arthritis (referred to self-reported arthritis henceforth) was defined by asking participants whether they ever received a diagnosis from a health professional for rheumatoid arthritis, gout, lupus, fibromyalgia, or some other form of arthritis. Since 2002, self-reported doctor-diagnosed arthritis has been recommended by the Arthritis Program at the Centers for Disease Control and Prevention for use in studies and health surveillance systems.

#### SES domains

There is some evidence that maternal education, paternal occupation and the financial situation of the family adequately reflect childhood SES as it relates to health [27]. In the absence of data on parental income or accumulated wealth, we used parental homeownership status at the time of childhood as an indicator of financial situation, considering the role of homeownership as the foremost asset-management avenue in the United States. We classified paternal occupation according to U.S. Census codes [28], dichotomized into high-SES occupations (“management, professional, and related occupations”; “sales and office occupations”) and low-SES occupations (“Service occupations”, “farming, fishing, and forestry occupations”, and “production, transportation, and material moving occupations”). Likewise, we dichotomized maternal education into high-SES (high school or more) and low-SES categories (less than high school). We used educational or occupational information on the other parent or the designated caretaker for participants with missing data on maternal education (n = 26 [3.3%]) or paternal occupation (n = 36 [4.6%]). Participants were asked to describe their living arrangements during childhood, and parental homeownership was classified as high-SES if the parents or caretakers were homeowners and as low-SES otherwise.

We assessed the current SES of participants using their own education, occupation and homeownership status, so that similar socioeconomic dimensions characterized SES at both life stages. Current socioeconomic characteristics were categorized according to a higher threshold than parental ones in order to account for the shift towards the tertiary economic sector and for the substantial increase in high-school graduation and college attendance rates during the past century. We therefore dichotomized “management, professional, and related occupations” occupations and levels of education greater than high school as high-SES, relegating “Sales and office” occupations and high-school level of education to the low-SES category.

#### Covariates

Body mass index (continuous BMI, in kg/m^2^), age (continuous years, at time of survey), and gender (0: male [referent], 1: female) have established links with the development of arthritis [8] and were included in our analyses. There is ambivalent evidence for a lower prevalence for arthritis among African-Americans [29-31], and we further adjusted our analyses for self-reported race (0: Caucasian [referent], 1: African-American).

### Analyses

Lifecourse epidemiological research is traditionally articulated around three main hypotheses, namely that the timing of socioeconomic conditions determines their relation to later health (critical-phase), that low SES affects health proportionally to its duration (accumulation), and that changes in SES constitute the exposure associated with health (social mobility) [32]. In this study, we investigated cumulative but separate effects of current and childhood SES, first across socioeconomic domains and then using aggregate SES scores. We further looked into the specific impact of each trajectory from childhood to current SES scores.

### SES summary scores

We created childhood and current SES summary scores for every participant by enumerating their low-SES characteristics. Not receiving any formal education past high school, not being a homeowner, and listing a low-SES occupation each increased a participant’s current SES score by 1; likewise, maternal education below high-school, low-SES paternal occupation and parental non-homeownership incremented childhood SES scores by 1. Summary scores were categorized as high (0 [referent]), medium (1), low (2), or lowest (3); for instance, a participant with a “production, transportation, and material moving” occupation with an associate degree and currently owning his or her home would have reached the medium category of current SES (1 + 0 + 0); a respondent whose mother did not complete high-school, whose father held a “sales or office” occupation and whose parents were not homeowners would have received a low childhood SES score (1 + 0 + 1).

Finally, we created an SES trajectory variable with categories for every possible permutation of childhood and current SES scores (e.g. high childhood SES/medium current SES, medium/low, high/high). For this purpose, “low” and “lowest” SES scores categories were collapsed together in order to reduce the total number of categories from 16 down to 9 and maintain meaningful cell sizes. A participant with all aforementioned socioeconomic characteristics would have fit in the low/medium trajectory.

### Statistical analyses

All statistical analyses were performed using Stata version 11.0 (StataCorp LP, College Station, TX). All tests of statistical significance are two-sided and considered significant at the 0.05 level. Univariate analyses were carried out to describe sociodemographic and health characteristics for the entire sample, and for participants with and without arthritis.

We evaluated associations between self-reported arthritis and SES using covariate adjusted logistic regressions across three approaches, as follows: (1) SES domains: for each domain of SES, participant and parental characteristics were included together in unadjusted models successively adding gender and race, then age and then BMI; (2) SES summary scores: categories of current and childhood SES summary scores were separately included in unadjusted models, and then together with progressive adjustments for race and gender, then age, then BMI; (3) SES trajectories: all 9 SES trajectories were included in a fully-adjusted model. Regression models were clustered by NC-FM-RN practitioner site and used the category of highest SES as the referent level. With significant covariate-adjusted associations of SES with self-reported arthritis, the mediation effect of BMI was assessed following the method proposed by Imai and colleagues [33]. Analyses in approach 2 and 3 were repeated including ordinal SES scores instead of categories thereof, in order to assess the significance of trends within our models.

Due to the complex relationships between race, gender and SES, we tested for effect measure modification by adding race or gender interaction terms to fully-adjusted regressions separately, including each SES variable and composite scores. We detected significant interactions of race with parental education (p = 0.044), and of gender with parental homeownership (p = 0.004), participant occupation (p = 0.022), and with the medium category of current SES (p = 0.013). None of these interaction effects remained significant upon correction using the Bonferroni criterion (adjusted significance level: p < 0.003), and we therefore did not stratify our sample according to gender or race.

## Results

### Participant characteristics

The sociodemographic and health characteristics of the entire sample and according to self-reported arthritis status can be found Table 1. Our sample was predominantly female (71%) and Caucasian (83%), and the mean age was 57 years old (range: 22–94). Three-fourths of all participants were overweight, with the mean BMI just under the obesity threshold (29.5 kg/m^2^), and 60% reported having doctor-diagnosed arthritis.

**Table 1:** Participant characteristics for the entire sample and for individuals with and without self-reported doctor-diagnosed arthritis

	**Entire sample**	**Arthritis**	**No arthritis**
	**(n = 1276)**	**(n = 767)**	**(n = 509)**
**Key variable**	**N (%) or**	**N (%) or**	**N (%) or**
	**mean (S.D.)**	**mean (S.D.)**	**mean (S.D.)**
Female	899 (70)	561 (73)	338 (66)
African-American	200 (16)	117 (15)	83 (16)
Age (22–94)	57.0 (14.0)	60.1 (12.8)	52.3 (14.5)
BMI^1^ (13–65)	29.5 (6.8)	30.3 (6.9)	28.4 (6.3)
*Socioeconomic characteristics*			
Non-homeowner^†^	216 (17)	134 (17)	82 (16)
*Education*			
> High School	817 (64)	445 (58)	372 (73)
High School^†^	327 (26)	218 (28)	109 (21)
< High School^†^	132 (10)	104 (14)	28 (6)
*Occupation*			
Professional/managerial	540 (42)	292 (38)	248 (49)
Sales and office^†^	287 (22)	173 (23)	114 (22)
Other^†^	449 (35)	302 (39)	147 (29)
*Childhood socioeconomic characteristics*			
Parents not homeowners^‡^	343 (73)	233 (30)	110 (22)
*Maternal Education*			
> High School	258 (20)	131 (17)	127 (25)
High School	413 (32)	218 (28)	195 (38)
< High School^‡^	605 (47)	418 (54)	187 (37)
*Paternal occupation*			
Professional/managerial	274 (21)	156 (20)	118 (23)
Sales and office	117 (9)	60 (8)	57 (11)
Other^‡^	885 (69)	551 (72)	334 (66)
*SES summary scores*			
*Current*^*2*^			
High	426 (33)	222 (29)	204 (40)
Medium	388 (30)	227 (30)	161 (32)
Low	363 (28)	250 (33)	113 (22)
Lowest	99 (8)	68 (9)	31 (6)
*Childhood*^*3*^			
High	268 (21)	134 (17)	134 (26)
Medium	395 (31)	218 (28)	177 (35)
Low	401 (31)	261 (34)	140 (28)
Lowest	212 (17)	154 (20)	58 (11)
*SES Trajectories (childhood to current)*^*4*^			
High to high	162 (13)	76 (10)	86 (17)
High to medium	73 (6)	38 (5)	35 (7)
High to low/lowest	33 (3)	20 (3)	13 (3)
Medium to high	154 (12)	81 (11)	73 (14)
Medium to medium	139 (11)	73 (10)	66 (13)
Medium to low/lowest	102 (8)	64 (8)	38 (7)
Low/Lowest to high	110 (9)	65 (8)	45 (9)
Low/Lowest to medium	176 (14)	116 (15)	60 (12)
Low/Lowest to low/lowest	327 (26)	234 (31)	93 (18)

^1^Body mass index, in kg/m^2^.

^2^Count of current low-SES (^†^) characteristics.

^3^Count of childhood low-SES (^‡^) characteristics.

^4^Permutations of childhood and current summary SES categories; “low” and “lowest” summary scores collapsed together.

Most participants were homeowners (83%), nearly half (42%) of them held professional or managerial occupations, and two-thirds received some formal education past high-school. Despite the more stringent dichotomization of education and occupation for participants, current SES scores were lower (greater SES) than childhood SES ones; 36% of sample participants either had a ‘low’ or ‘lowest’ current SES, whereas nearly half (48%) were in the lower two categories of childhood SES. The number of individuals whose SES fell from ‘high’ during childhood to ‘low’ or ‘lowest’ in adulthood was especially small (n = 33, 3%).

Self-reported arthritis was somewhat more prevalent among women (62%) than among men (55%), and affected a similar proportion of Caucasians (60%) and African Americans (58%). Compared to undiagnosed respondents, participants with self-reported arthritis had a higher BMI, were older, less educated, less likely to hold a professional or managerial occupation, and were raised in lower-SES households according to all three SES domains.

### Regression results

#### Approach 1: SES domains

In unadjusted models separately including SES domains at both life-stages (Table 2), participant (OR = 1.61 [95%CI = 1.29, 2.02]) and parental education (OR = 1.78 [95%CI = 1.36, 2.31]) were significantly associated with the odds of reporting arthritis. Associations with homeownership and with occupation were confined to either life-stage, with low-SES participant occupation (OR = 1.48 [95% CI = 1.13, 1.93]) and parental homeownership (OR = 1.58 [95%CI = 1.28, 1.95]) being associated with greater odds for self-reported arthritis. Progressive adjustments for covariates lowered parameter estimates for both participant and parental education, and for parental homeownership, but did not drop them below the significant level. BMI, when introduced in gender, race and age-adjusted models, explained 20% (95% CI = 11%, 73%) of the remaining association of low parental education with arthritis, and 25% (95% CI = 16%, 57%) of the influence of parental non-homeownership (data not shown).

**Table 2:** **Associations**^**1 **^**of childhood and current socioeconomic characteristics with odds of reporting doctor-diagnosed arthritis**^**2**^

	**Adjusted for:**				
**SES domains (N)**	**Unadjusted**	**Gender**	**Gender, race**	**Gender, race,age**	**Gender, race, age, bmi**
Education					
Low current (459)	**1.61 (1.29, 2.02)**	**1.60 (1.28, 2.01)**	**1.67 (1.34, 2.09)**	**1.62 (1.28, 2.05)**	**1.53 (1.19, 1.97)**
Low parental (605)	**1.78 (1.36, 2.31)**	**1.75 (1.33, 2.31)**	**1.82 (1.39, 2.37)**	**1.37 (1.07, 1.76)**	**1.30 (1.01, 1.66)**
Occupation					
Low current (736)	**1.48 (1.13, 1.93)**	**1.48 (1.13, 1.93)**	**1.51 (1.16, 1.97)**	**1.49 (1.11, 2.00)**	**1.49 (1.14, 1.94)**
Low parental (885)	1.20 (0.93, 1.56)	1.18 (0.90, 1.55)	1.21 (0.92, 1.60)	1.16 (0.89, 1.52)	1.06 (0.82, 1.38)
Homeownership					
Low current (216)	1.02 (0.68, 1.54)	0.99 (0.65, 1.50)	1.02 (0.68, 1.55)	1.28 (0.81, 2.02)	1.27 (0.79, 2.03)
Low parental (343)	**1.58 (1.28, 1.95)**	**1.55 (1.26, 1.91)**	**1.57 (1.28, 1.93)**	**1.40 (1.17, 1.67)**	**1.29 (1.07, 1.56)**

Bolded results are significant at the 0.05 level.

Socioeconomic domains included separately.

^1^Parameters are odds ratio (OR) and 95% confidence intervals (CI).

^2^Referent categories are respective high-SES alternatives.

#### Approach 2: SES summary scores

Categories of current and childhood SES scores were included in univariate logistic models, and then together with progressive adjustments for gender and race, age, and then BMI (Table 3). In univariate regressions, lower levels of childhood and current SES were associated with incrementally greater odds of arthritis reporting, with childhood SES having a somewhat larger influence than current SES (lowest childhood SES OR = 2.66 [95%CI = 1.22, 3.32] vs. lowest current SES OR = 2.02 [95%CI = 1.92, 3.68]). The association of current SES with the reporting of arthritis was considerably weakened when current and childhood scores were simultaneously including (Model 1), and it only remained significant in the low (but not lowest) category.

**Table 3:** **Associations of childhood and current socioeconomic status summary scores with odds of having self-reported doctor-diagnosed arthritis**^**1**^

**SES Scores (N)**	**Univariate**	**Model 1**	**Model 2**	**Model 3**	**Model 4**
	**OR (95% CI)**	**OR (95% CI)**	**OR (95% CI)**	**OR (95% CI)**	**OR (95% CI)**
Current^2^					
High (426)	1.00	1.00	1.00	1.00	1.00
Med (388)	**1.30 (1.04, 1.61)**	1.17 (0.91, 1.50)	1.19 (0.92, 1.53)	1.29 (0.98, 1.71)	1.28 (0.98, 1.66)
Low (363)	**2.03 (1.47, 2.81)**	**1.62 (1.16, 2.25)**	**1.67 (1.22, 2.30)**	**1.71 (1.16, 2.51)**	**1.66 (1.14, 2.42)**
Lowest (99)	**2.02 (1.22, 3.32)**	1.49 (0.95, 2.34)	**1.68 (1.08, 2.62)**	**2.11 (1.21, 3.67)**	**2.08 (1.16, 3.74)**
Childhood^3^					
High (268)	1.00	1.00	1.00	1.00	1.00
Med (395)	**1.23 (1.01, 1.50)**	1.14 (0.92, 1.42)	1.17 (0.94, 1.46)	1.10 (0.86, 1.41)	1.02 (0.80, 1.31)
Low (401)	**1.86 (1.37, 2.53)**	**1.58 (1.15, 2.17)**	**1.66 (1.21, 2.28)**	1.26 (0.91, 1.75)	1.14 (0.82, 1.59)
Lowest (212)	**2.66 (1.92, 3.68)**	**2.13 (1.56, 2.90)**	**2.19 (1.58, 3.05)**	**1.62 (1.20, 2.19)**	**1.39 (1.04, 1.85)**

Bolded results are significant at the 0.05 level.

Model 1: together, without covariate adjustments; Model 2: mutually adjusted for race gender; Model 3: mutually adjusted for race, gender, age; Model 4: mutually adjusted for race, gender, age, bmi.

^1^Parameters are odds ratio (OR) and 95% confidence intervals (CI).

^2^Current SES referent category defined as some education past high school, homeownership and occupation either “professional” or “managerial and related”.

^3^Childhood SES referent category defined as maternal education greater than or equal to than high school, paternal occupation “professional”, “managerial and related” or “sales and office”, and parental homeownership.

Adjustments for gender and race (Model 2) restored the association of lowest current SES with self-reported arthritis (OR = 1.68 [95% CI = 1.08, 2.62]). Adding age to the model (Model 3) weakened the influence of childhood SES, now limited to the lowest category (OR = 1.62 [95% CI = 1.20, 2.19]), while increasing parameter estimates for current SES, at both the low (OR = 1.71 [95% CI = 1.16, 2.51]) and the lowest levels (OR = 2.11 [95% CI = 1.21, 3.67]). Finally adding BMI (Model 4) explained a third (33% [95% CI = 21%, 84%] of the remaining influence of lowest childhood SES (data not shown) while marginally affecting current SES (low SES: 9% mediated; lowest SES: 6% mediated).

#### Approach 3: SES trajectories

All 9 possible categories of SES trajectory were included in a covariate-adjusted model (Table 4). Relative to the high/high referent category, the lowest three combinations of childhood and current SES (low/low, low/medium, and medium/low) were associated with significantly elevated odds of having self-reported arthritis. The relationship was strongest among participants whose SES fell from ‘medium’ in childhood to ‘low’ in adulthood (OR = 2.20 [95% CI: 1.29, 3.75]), followed by those with a consistently ‘low’ SES (OR = 2.05 [95% CI: 1.35, 3.12]), and then by individuals whose SES rose from the ‘low’ category to the ‘medium’ level (OR = 1.76 [95% CI: 1.15, 2.70]). The odds of reporting an arthritis diagnosis were nearly twice greater in the high-to-low trajectory than among referent participants (OR = 1.93 [95% CI: 0.85, 4.38]); however, this result was not statistically significant, likely due to the small number of respondents experiencing such a drop in SES (n = 33). Significant trends of increasing self-reported arthritis odds with worsening current SES could be observed within each category of childhood SES (p < 0.05), whereas the influence of childhood SES within each level of current SES was not statistically significant.

**Table 4:** **Odds ratio and 95**% **confidence intervals for the associations of trajectories**^**1 **^**of socioeconomic status**^**2 **^**with odds of having self-reported doctor-diagnosed arthritis**^**3**^

	**Current SES:**		
	**High**	**Medium**	**Low/Lowest**
	(n = 426)	(n = 388)	(n = 462)
Childhood SES: High (n = 268)	1.00	1.40 (0.83, 2.36)	1.93 (0.85, 4.38)
	n = 162	n = 73	n = 33
Medium (n = 395)	1.09 (0.72, 1.63)	1.15 (0.82, 1.60)	**2.20 (1.29, 3.75)**
	n = 154	n = 139	**n = 102**
Low/Lowest (n = 613)	1.26 (0.83, 1.93)	**1.76 (1.15, 2.70)**	**2.05 (1.35, 3.12)**
	n = 110	**n = 176**	**n = 327**

Bolded results are significant at the 0.05 level.

Model 1: adjusted for age, gender, race; Model 2: adjusted for age, gender, race, bmi.

^1^‘Lowest’ and ‘Low’ summary scores collapsed into ‘low’ category.

^2^Referent category defined as ‘high’ current SES and ‘high’ childhood SES, meaning: education strictly greater than high school, homeownership, occupation other than “professional” or “managerial and related”, maternal education greater than or equal to high school, paternal occupation qualified as “professional”, “managerial and related” or “sales and office”, and parental homeownership.

3 Parameters are odds ratio (OR) and 95% confidence intervals (CI).

## Discussion

Our results suggest that low SES in childhood and in later life cumulatively add to the risk of developing arthritis, following a gradient pattern of increased risk at lower SES levels. SES in later life more closely aligned with self-reported arthritis than childhood SES did, and there was limited evidence that a currently favorable SES may mitigate the impact of previously adverse circumstances.

While analyses based on observational data have limited power to establish causal relations, BMI appeared to be a credible pathway linking the lowest childhood SES category and later arthritis onset, with a third of the demographics-adjusted association explained by the elevated BMI of participants reared in disadvantaged environments. This is consistent with the established role of weight in arthritis etiology – especially OA, and with mounting evidence that childhood SES enduringly influences adult BMI [34]. The self-reported, doctor-diagnosed definition for arthritis encompasses diverse, etiologically complex disorders, and a multitude of further mechanisms potentially contributed to our results. Unmeasured socioeconomically-patterned health-behaviors taking their roots across life-stages could likely explain some of our findings, including cigarette-smoking, inadequate physical activity and diet composition [15,35-38]. Our results may also reflect the increased exposure of low-SES individuals to long-term environmental risk factors, such as infection and musculoskeletal injury [14,20,22,39,40], and to psychosocial adversity. Strong evidence notably suggests a link between the experience of childhood adversity and a person’s susceptibility to arthritis independent of health behaviors [17,41,42]. Of further note, and perhaps underlying the above mechanisms, socioeconomic differences in immune functioning may lastingly increase the vulnerability of low-SES individuals to inflammatory disorders such as arthritis [16,43].

Studies of socioeconomic disparities in the rheumatic diseases often limit their analyses to single domains of SES, typically education or occupation. Interestingly, our results were quite sensitive to the domain of SES used when each was taken separately, to the extent that analyses solely using education, occupation or homeownership could support three distinct conclusions (Table 3), respectively that self-reported arthritis is associated with both current and childhood SES, current SES only, or childhood SES only. This suggests that SES domains do not interchangeably correlate with arthritis onset, perhaps due to their varying associations with risk-factors: for instance, education directly informs and shapes health-behaviors, while occupation may more closely capture risk-factors in the workplace such as physically-intensive work.

The prevalence of self-reported arthritis was similar among Caucasians and African-Americans participants; however, the latter had meaningfully lower-odds of reporting arthritis once SES was accounted for (data not shown). This was most evident in fully-adjusted approaches simultaneously including categories of current and childhood SES scores (OR = 0.65 [95% CI = 0.52, 0.81]), and least so when homeownership alone modeled SES (OR = 0.75 [95% CI = 0.59, 0.95]). These findings were consistent with data from the Johnston County cohort (North Carolina). In cross-sectional analyses not adjusted for SES variables, the investigators reported similar risks of hip and knee OA according to race [29,44]; however, African-Americans were less likely to develop Hip-OA than Caucasians in longitudinal analyses adjusted for education (Hip: OR = 0.44), although they experienced more severe disease [45].

The main strengths of this study are that it examines the associations of SES with self-reported arthritis within a large group of individuals from practices representative of North Carolina, and uses several analytic approaches involving multiple domains of SES, adjusting for key confounders, and evaluating BMI as a potential pathway. Several methodological weaknesses should be taken into account in the interpretation of our results. First instance, our recruitment from family-practices excluded individuals who did not visit a primary-care provider, perhaps due to socioeconomic reasons. Like all self-reported measures, reporting an arthritis diagnosis may reflect various non-clinical factors, such as the access to and utilization of health services needed to receive a professional opinion [46-48]. The relationship between SES, health perceptions and reporting behaviors may likewise bias our findings, although the direction and magnitude of these effects remain uncertain [49,50]; so far, two validation studies conducted by the CDC did not find significant differences in the sensitivity or specificity of the BRFSS self-reported arthritis according to education [51,52].

Our use of recollected parental socioeconomic characteristics inevitably introduced error to our analyses. Previous studies found such measures to be reliable or optimistic representations of parental SES [53], which could downplay the influence of early-SES on later health. Additionally, cross-sectional studies are vulnerable to reverse-causality. In our case, the economic burden of arthritis could threaten an individual’s career (occupation) and ability to own a home, and the heritability of arthritis disorders may hypothetically link low parental SES due to arthritis with disease onset among participants; however, the latter was likely limited by our use of recalled parental characteristics predating the development of most arthritis types, and the educational level of participants, most closely aligned with self-reported arthritis in our analyses, was likely established prior to arthritis onset.

## Conclusions

This study provides evidence that socioeconomic disparities in the prevalence of arthritis take their roots early in the lifecourse, although the stronger and possibly superseding influence of current SES must be noted. BMI acted as a meaningful pathway linking childhood SES with self-reported arthritis, and efforts aimed at combating overweightness among disadvantaged children may viably mitigate some of the musculoskeletal health disparities arising in later life. To the extent that health inequalities underscore preventable risk-factors, efforts towards reducing the prevalence of arthritis would gain from the study of further pathways connecting SES across life stages and arthritis etiology, with smoking, inadequate physical activity and musculoskeletal injuries being plausible contenders.

Further studies on socioeconomic differences in the prevalence of arthritis should include diverse domains of SES measured at different life-stages. This study’s generalizability may be limited outside of its geographic (North Carolina) and demographic (59% white females) boundaries, and its findings need to be replicated in other populations. It would be of particular interest to identify whether disparities in arthritis risk are specific to given socio-demographic contexts. For instance, gender-specific associations between BMI and SES may plausibly give rise to different patterns of musculoskeletal-health inequalities in men and women. Moreover, the intricate relationship between race, SES and health warrants further investigations within racial groups, especially in light of the arresting relationship between race and self-reported arthritis observed here and in previous research [45].

## Consent

All participants provided written consent for their involvement in this study.

## Abbreviations

BMI: Body mass index; NC-FM-RN: North Carolina family-medicine research network; OA: Osteoarthritis; RA: Rheumatoid arthritis; SES: Socioeconomic status; SODE: Individual and community social determinants of arthritis outcomes study.

## Competing interests

The authors declare that they have no competing interests.

## Authors’ contributions

AB participated in study conception and design, performed statistical analyses, and drafted the manuscript; RC participated in statistical analyses, study conception and design, and helped draft the manuscript; LC participated in data collection, study conception and design, and helped draft the manuscript. All authors read and approved the final manuscript.

## Pre-publication history

The pre-publication history for this paper can be accessed here:

http://www.biomedcentral.com/1471-2474/14/327/prepub
